# HIV-1 Vpu Promotes Release and Prevents Endocytosis of Nascent Retrovirus Particles from the Plasma Membrane

**DOI:** 10.1371/journal.ppat.0020039

**Published:** 2006-05-12

**Authors:** Stuart J. D Neil, Scott W Eastman, Nolwenn Jouvenet, Paul D Bieniasz

**Affiliations:** Aaron Diamond AIDS Research Center and Laboratory of Retrovirology, Rockefeller University, New York, New York, United States of America; King's College London, United Kingdom

## Abstract

The human immunodeficiency virus (HIV) type-1 viral protein U (Vpu) protein enhances the release of diverse retroviruses from human, but not monkey, cells and is thought to do so by ablating a dominant restriction to particle release. Here, we determined how Vpu expression affects the subcellular distribution of HIV-1 and murine leukemia virus (MLV) Gag proteins in human cells where Vpu is, or is not, required for efficient particle release. In HeLa cells, where Vpu enhances HIV-1 and MLV release approximately 10-fold, concentrations of HIV-1 Gag and MLV Gag fused to cyan fluorescent protein (CFP) were initially detected at the plasma membrane, but then accumulated over time in early and late endosomes. Endosomal accumulation of Gag-CFP was prevented by Vpu expression and, importantly, inhibition of plasma membrane to early endosome transport by dominant negative mutants of Rab5a, dynamin, and EPS-15. Additionally, accumulation of both HIV and MLV Gag in endosomes required a functional late-budding domain. In human HOS cells, where HIV-1 and MLV release was efficient even in the absence of Vpu, Gag proteins were localized predominantly at the plasma membrane, irrespective of Vpu expression or manipulation of endocytic transport. While these data indicated that Vpu inhibits nascent virion endocytosis, Vpu did not affect transferrin endocytosis. Moreover, inhibition of endocytosis did not restore Vpu-defective HIV-1 release in HeLa cells, but instead resulted in accumulation of mature virions that could be released from the cell surface by protease treatment. Thus, these findings suggest that a specific activity that is present in HeLa cells, but not in HOS cells, and is counteracted by Vpu, traps assembled retrovirus particles at the cell surface. This entrapment leads to subsequent endocytosis by a Rab5a- and clathrin-dependent mechanism and intracellular sequestration of virions in endosomes.

## Synopsis

Human immunodeficiency virus (HIV) type-1 encodes a small protein, termed HIV-1 viral protein U (Vpu), which enhances the release of retroviral particles from certain cell types. This study shows that Vpu affects the distribution of the major retroviral structural protein within cells. In particular, the viral structural protein was initially detected at the exterior surface of the cell, where it assembles into particles, but over time was internalized. Vpu prevented internalization, and instead induced the release of particles from the cell surface. The effect of Vpu was specific for retroviral particles and simply blocking particle internalization did not obviate the requirement for Vpu. These experiments strongly suggest that Vpu counteracts a cellular activity that traps newly formed retrovirus particles at the cell surface. Surface entrapment of retroviral particles ultimately leads to their internalization to the cell interior, rather than their release to infect new cells.

## Introduction

Human immunodeficiency virus (HIV) type-1 viral protein U (Vpu) is a small (16 kDa) membrane protein encoded by HIV-1 [1,2] and certain simian immunodeficiency viruses with which HIV-1 likely shares a common ancestor [3–7]. Vpu appears to have two major functions in HIV-1 replication (reviewed in [8]). First, it associates with newly synthesized CD4 in the endoplasmic reticulum and recruits βTrCP/SCF ubiquitin ligases to mediate its degradation by proteosomes [9], perhaps following dislocation [10]. This is thought to prevent CD4–Env binding in the endoplasmic reticulum [11,12], and thereby to facilitate proper Env assembly into virions, and perhaps prevent retention of virions at the cell surface via CD4–Env interactions [13–15]. Second, Vpu expression enhances HIV-1 particle release by an additional, ill-defined, Env- and CD4-independent mechanism [16–19]. Vpu-defective HIV-1 mutants replicate poorly in CD4+ T cells and macrophages [8,17], and recent studies in macaques have demonstrated that Vpu-defective simian–HIV strains are attenuated in vivo [20].

Structurally, Vpu consists of two major domains: an N-terminal transmembrane (TM) domain that anchors Vpu in cellular membranes and appears to form a cation channel [21,22], and a cytoplasmic tail consisting of two putative α-helices separated by a conserved casein kinase II phosphorylation site [23]. Both the cytoplasmic tail and the TM domain are required for CD4 down-regulation [24]. Conversely, while truncations of the cytoplasmic tail are reported to have little effect on the ability of Vpu to enhance virion release, mutations that truncate the TM domain or change blocks of conserved residues within it impede this second function [24–26].

Early studies showed that expression of Vpu could enhance the release of diverse retroviral particles from human cells, irrespective of whether those retroviruses normally encode a Vpu protein [18]. More recent studies strongly suggest that Vpu, as well as the Vpu-like activity of HIV-2 envelope proteins [27], acts by overcoming a novel species-specific host restriction to HIV release [28,29]. Specifically, while efficient HIV-1 particle release from African green monkey (AGM) COS cells exhibits no requirement for Vpu expression, release from heterokaryons formed between COS and various human cell lines appears Vpu-dependent [28]. Additionally, the Env protein of HIV-2, previously reported to harbor a Vpu-like activity in that it stimulates virion release [27], also enhances HIV-1 and murine leukemia virus (MLV) release from simian–human cell heterokaryons [29]. The host factors responsible for this apparently dominant restriction and the method by which they act to inhibit release are undefined at present. Nonetheless, several electron microscopy studies showed that Vpu-defective HIV-1 accumulates as morphologically mature virion particles at the plasma membrane (PM) and in intracellular vacuoles [17,18]. In addition, one previous study suggested that Vpu changes the subcellular localization of HIV-1 Gag [30].

The matrix domain of HIV-1 Gag is largely responsible for targeting to the cellular membrane, but conflicting studies have postulated a role for HIV-1 matrix in Vpu-mediated particle release [31,32]. Nonetheless, the appearance of HIV-1 Vpu-defective particles in internal membrane-bound compartments [17,18] is suggestive of a Gag targeting defect, or a defect in the endosome-to-PM trafficking pathway that has recently been proposed to be important for retrovirus assembly and release [33–41]. Importantly, the HIV-1 morphogenesis defect associated with Vpu deficiency is distinct from that associated with p6 mutations that abolish late-budding domain (L-domain) function [42]. In the latter case, class E vacuolar protein-sorting (VPS) factor recruitment is defective, resulting in maintenance of continuity between viral and cellular membrane and accumulation of incompletely formed immature virions at the PM [43,44].

In this study, we determined the effects of Vpu on HIV-1 and MLV Gag distribution in cells where Vpu is required, or not required, for efficient virion release. Notably, we find that Vpu can cause a dramatic change in HIV-1 and MLV Gag localization in cells where it enhances virus release. Specifically, Vpu prevented accumulation of HIV-1 and MLV Gag in endosomal compartments. Interestingly, inhibition of endocytosis or early endosome function similarly prevented the intracellular accumulation of Gag in endosomes, but did not rescue Vpu-defective virus release. Moreover, Gag localization to endosomes required that it be competent to complete late-budding steps. Thus, we propose that Vpu overcomes a host activity that prevents the release of completely formed virions from the cell surface, and that this host cell–specific activity leads to subsequent virion endocytosis and sequestration in endosomes.

## Results

### Vpu-Mediated Enhancement of Retroviral Release Is Human Cell Type–Dependent

**Figure 1:**
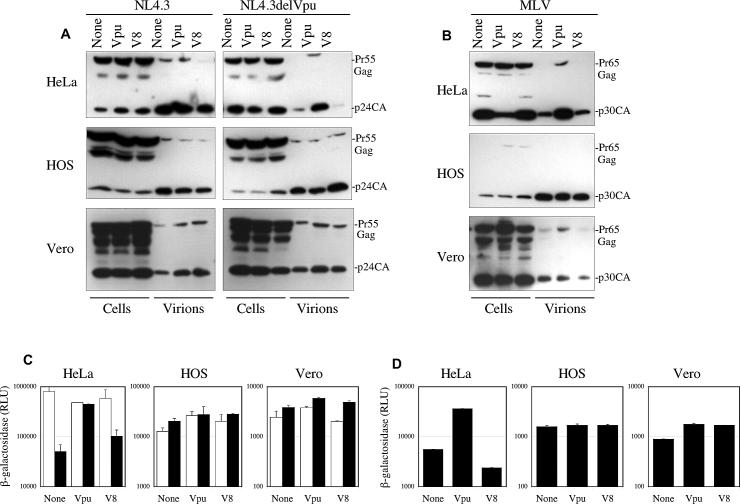
Cell Type–Dependent Effects of Vpu on Retroviral Particle Release Human HeLa and HOS cells, together with AGM Vero cells, were transfected with HIV-1 NL4.3 and NL4.3delVpu proviral plasmids, or an MLV Gag-Pol–expression vector, as indicated, in the presence of plasmids expressing either no Vpu (none), codon-optimized Vpu, or an inactive Vpu mutant containing a CD8 TM domain (V8). (A and B) Western blot analyses of cell and virion lysates, probed using antibodies to HIV-1 (A) or MLV CA (B). (C and D) Results of chemiluminsecent β-galactosidase assays following inoculation of HeLa-TZM indicator cells with supernatants derived from HeLa, HOS, or Vero cells transfected with NL4.3 (white bars) or NL4.3delVpu (black bars) proviral plasmids (C) or plasmids expressing MLV Gag-Pol, a packageable HIV-1 Tat–expression vector, and VSV-G (D). Vpu and control (GFP)–expression plasmids were included, as indicated, and results plotted as relative light units (RLU) ± standard deviation of the mean.

To permit studies of the effects of Vpu on retrovirus assembly and release, we first sought to identify human cell lines in which particle release was Vpu-dependent and -independent. Cells were transfected with an HIV-1 proviral plasmid (NL4.3), a Vpu-defective derivative (NL4.3delVpu), or an MLV Gag-Pol–expression plasmid along with plasmids expressing green fluorescent protein (GFP, as a control), Vpu, or an inactive Vpu mutant in which the TM domain was replaced by that of human CD8 (V8). As expected, Western blot and infectious virion titration assays showed that HIV-1 release from human HeLa cells was clearly responsive to Vpu (Figure 1). Specifically, the levels of extracellular virions, as detected by an anti-p24CA antibody, from cells transfected with the NL4.3delVpu proviral plasmid, were significantly reduced as compared to those released from wild-type proviral plasmid-transfected cells, despite equivalent levels of precursor p55Gag levels in the cell lysates (Figure 1A). Expression of Vpu in *trans* rescued Vpu-defective HIV-1 release to levels similar to those of the wild-type virus, whereas the mutant Vpu, V8, was inactive (Figure 1A).

As has previously been shown [18,29], Vpu also stimulated MLV particle release from HeLa cells (Figure 1B). In the case of MLV, it was notable that more mature processed capsid (CA, p30) accumulated in HeLa cells in the absence of Vpu than in its presence, despite unchanged levels of Pr65 Gag precursor (Figure 1B). Thus, Vpu expression apparently reduced HeLa cell–associated mature MLV CA and redistributed it to the culture supernatant. Infectious virion–release assays also showed that Vpu expression enhanced NL4.3delVpu release from HeLa cells by approximately 10-fold, as assayed by β-galactosidase activity in TZM indicator target cells (Figure 1C). A similar increase in yield of infectious MLV vector particles (pseudotyped with VSV-G and carrying an HIV-1 Tat–expressing vector) was obtained in response to Vpu expression in virus-producing HeLa cells (Figure 1D).

In contrast, the effect of Vpu on HIV-1 or MLV release was very different in HOS cells (Figure 1A–1D). Specifically, NL4.3 and NL4.3delVpu were released equivalently from HOS cells, and there was no discernable effect of Vpu expression in *trans* on the level of HIV-1 or MLV particle release, measured using Western blot or infectious particle assays (Figure 1A–1D). Curiously, when compared to HeLa cells, HOS cell lysates appeared to contain proportionally less HIV-1 CA compared to p55Gag (Figure 1A). Moreover, only low levels of mature or immature MLV Gag were detected in HOS cell lysates, despite reasonably abundant particle yield from culture supernatants (Figure 1B). These data suggested that mature HIV-1 and MLV particle release is somewhat more efficient from HOS cells than from HeLa cells. Finally, as a control, AGM Vero cells were also tested, and these results were consistent with previous studies that HIV-1 release from AGM cells is unresponsive to Vpu expression [28] (Figure 1A–1D). Taken together, these results show that the ability of Vpu to enhance retroviral release is not only dependent on host-cell species, as previously shown [28], but is also human cell type–dependent.

### HIV-1 and MLV Gag Accumulate in Endosomal Compartments in HeLa Cells but Not in HOS Cells, and This Accumulation Is Prevented by Vpu

**Figure 2:**
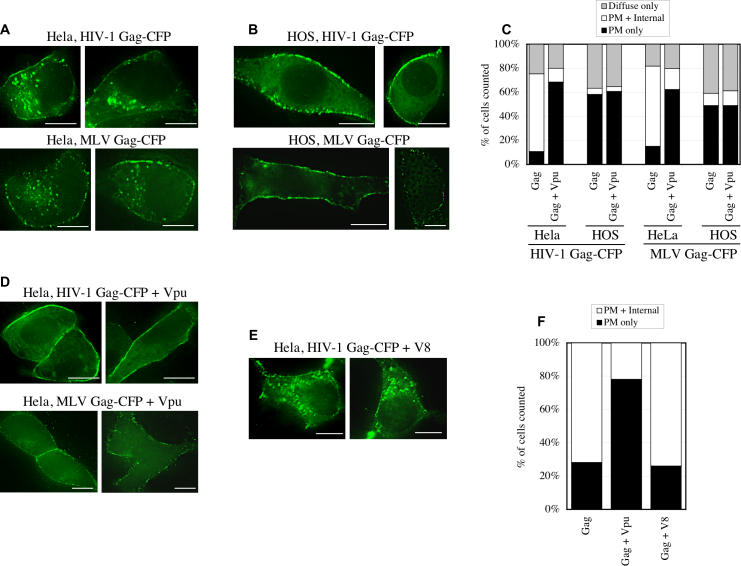
Expression of Vpu Prevents Intracellular Accumulation of Retroviral Gag-CFP Fusion Proteins in HeLa Cells Cells were transfected with the indicated expression vectors, fixed 18 h after transfection, and observed by deconvolution microscopy. (A and B) Localization of HIV-1 Gag-CFP or MLV Gag-CFP in HeLa cells (A) or HOS cells (B) in the absence of Vpu. Two examples of each cell type and each Gag-CFP protein are shown. White bars in the micrographs indicate a distance of 10 μm. (C) Quantitative analysis of these effects. Ten random fields of HeLa and HOS cells expressing HIV-1 or MLV Gag-CFP proteins in the presence or absence of Vpu were inspected, and the numbers of cells in which Gag-CFP was observed as accumulations at the PM only (black bars), at intracellular sites as well as at the PM (white bars), or as diffuse cytoplasmic fluorescence only (grey bars) were counted. Enumeration of cells with each pattern of Gag-CFP localization is expressed as a percentage the total of the cells counted, which was 75 ± 7 for each condition and is a representative example of three separate experiments. (D) Examples of HeLa cells expressing HIV-1 Gag-CFP (upper panels) or MLV Gag CFP (lower panels) in the presence of co-expressed Vpu. (E and F) The Vpu TM domain is required to prevent intracellular accumulation of HIV-1 Gag-CFP. (E) Examples of HeLa cells expressing HIV-1 Gag-CFP and V8. White bars in the micrographs indicate a distance of 10 μm. (F) Cells expressing HIV-1 Gag-CFP only, Gag-CFP and Vpu, or Gag-CFP and V8 were inspected and enumerated as described in (C), except that cells containing only diffuse Gag-CFP and no visible accumulations were excluded from the analysis.

Having identified human cell types (HeLa and HOS) in which Vpu expression differentially affected retroviral particle release, we next examined whether HIV-1 and MLV Gag proteins displayed any differences in distribution in HeLa and HOS cells. Cells were transfected with plasmids expressing HIV-1 or MLV Gag-CFP, then fixed and observed by deconvolution microscopy. Initially, observation was done using cells fixed at 18 h after transfection, and we found that HIV-1 and MLV Gag-CFP could exhibit three distinct patterns of localization in HeLa cells. First, a diffuse cytoplasmic fluorescence was observed primarily in cells expressing apparently lower levels of Gag-CFP (unpublished data). Alternatively, in cells expressing apparently higher levels of Gag-CFP, the fluorescent signal could be observed to be concentrated primarily either at the PM or in intracellular accumulations. Mixed distributions could also be observed and examples are shown in Figure 2A. In contrast, Gag-CFP only rarely exhibited discrete intracellular accumulations in HOS cells, with most cells exhibiting only PM-associated accumulation or low-level diffuse fluorescence (Figure 2B). By simply counting the number of Gag-CFP–positive cells on the basis of whether CFP accumulated only at the PM or at both the PM and intracellular compartments, we found that for both HIV-1 and MLV Gag-CFP, the majority of HeLa cells (~70%) exhibited clear intracellular accumulations of Gag-CFP, as well as PM fluorescence (Figure 2C), while less than 5% of HOS cells exhibited intracellular accumulations of Gag-CFP.

Notably, expression of Vpu along with Gag-CFP in HeLa cells dramatically reduced the number of cells containing intracellular Gag-CFP accumulations. This result was obtained with both HIV-1 Gag-CFP and MLV Gag-CFP (Figure 2C and 2D). In marked contrast, Vpu had no effect on HIV-1 or MLV Gag-CFP localization in HOS cells (Figure 2C; unpublished data). Importantly, the inactive mutant Vpu, V8, failed to prevent the occurrence of intracellular concentrations of HIV-1 Gag-CFP in HeLa cells (Figure 2E and 2F). Thus, the Vpu TM domain that is critical for virus release function [24] (Figure 1), is also required for effecting changes in HIV-1 and MLV Gag localization **(** Figure 2E and 2F), specifically in cells (HeLa) where Vpu is required for efficient virion release.

**Figure 3:**
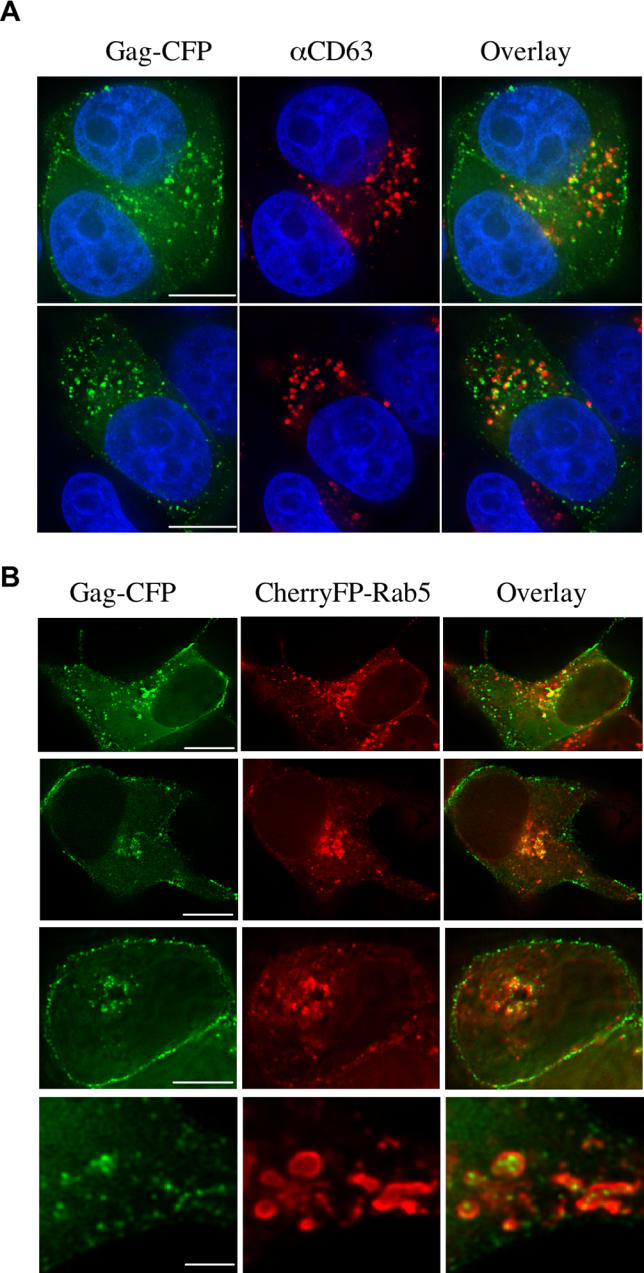
Intracellular HIV-1 Gag-CFP Partly Co-localizes with both Early and Late Endosomal Markers in HeLa Cells (A) HeLa cells expressing HIV-1 Gag-CFP (green) were fixed 18 h post-transfection and stained with a monoclonal antibody specific for human CD63, and a secondary anti-mouse Alexa-Fluor-588 conjugate (red). Nuclei were counter-stained with DAPI (blue). (B) HeLa cells expressing HIV-1 Gag-CFP (green) were co-transfected with CherryFP-Rab5a (red), a marker for early endosomal structures. Cells were fixed and images acquired at 18 h post-transfection. The lower set of images presented in (B) are shown at approximately 5-fold higher magnification to give a clearer indication of the juxtaposition of the HIV-1 Gag-CFP and CherryFP-Rab5a signals. White bars in the micrographs indicate a distance of 10 μm, except in the lower set of images in (B), where the bar indicates 2 μm

Intracellular accumulations of HIV-1 and MLV Gag have been proposed to be a consequence of Gag targeting and/or virion budding into late endosomal compartments [33–40]. Consistent with this, and as has been previously reported [34,36,37], some of the intracellular accumulations of HIV-1 Gag-CFP in HeLa cells were coincident with late endosomes as revealed by antibody staining for CD63 (Figure 3A). However, co-expression of HIV-1 Gag-CFP with a cherry fluorescent protein (CherryFP)–Rab5a fusion protein also revealed some degree of co-localization (Figure 3B). Rab5a is a GTPase that is responsible for regulating endocytic vesicle trafficking from the PM and the formation of early endosomes [45]. Thus, this result indicated that a proportion of HIV-1 Gag-CFP localizes to early endosomes (Figure 3B). In some cases, the Gag-CFP signal appeared to be surrounded by a CherryFP-Rab5a signal (Figure 3B, lower panels). A similar degree of co-localization with each of these endosomal markers was also observed with MLV Gag-CFP (unpublished data).

**Figure 4:**
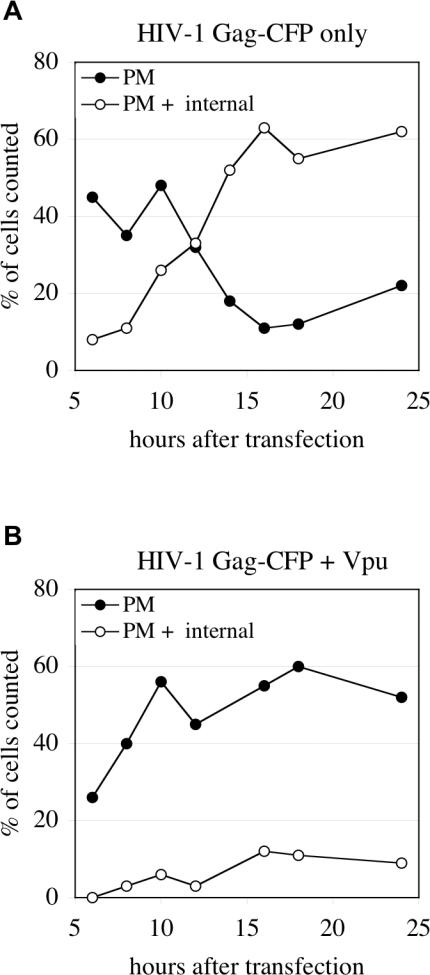
HIV-1 Gag-CFP Accumulation at the PM Precedes Accumulation at Intracellular Sites in HeLa Cells in the Absence of Vpu Expression (A and B) HeLa cells were transfected with HIV-1 Gag-CFP and either an irrelevant plasmid control (A) or a Vpu-expression plasmid (B). The cells were washed 5 h post-transfection and then fixed at 2-h intervals thereafter. The number of cells in ten fields that exhibited HIV-1 Gag-CFP accumulations at the PM only or at the PM and intracellular sites, or which expressed only diffuse Gag-CFP, were enumerated as in Figure 1C and plotted as the percentage of the total. Cells containing only diffuse Gag-CFP have been omitted from the charts for simplicity.

Based on this result, we hypothesized that Vpu might affect retroviral Gag trafficking through endosomes en route to the PM. In other words, in cells that require Vpu for efficient particle release, Gag molecules might be inappropriately targeted to endosomal compartments or retarded during transit through endosomal compartments. The likely consequence of such a scenario would be a failure or a delay in accumulation of Gag at the PM. To examine the kinetics of Gag accumulation at intracellular versus PM sites, we fixed HeLa cells at various times after transfection with an HIV-1 Gag-CFP–expression plasmid and counted the number of transfected cells that exhibited diffuse, PM, or internal Gag accumulations (Figure 4A). Surprisingly, visible accumulation of Gag-CFP at the PM significantly preceded visible accumulation at intracellular sites. Indeed, at early time points (<12 h after transfection) cells exhibiting Gag-CFP accumulations exclusively at the PM predominated and increased over time. However, at later time points (>12 h after transfection), the fraction of cells with significant intracellular Gag-CFP accumulations, in addition to PM fluorescence, increased (Figure 4A). Indeed, these cells eventually predominated and, by 16–18 h after transfection, an apparent steady state was achieved, with approximately 70% of the transfected cells containing primarily intracellular Gag-CFP accumulations. In marked contrast, co-expression of Vpu with Gag-CFP prevented the accrual of cells containing intracellular Gag-CFP accumulations, and cells exhibiting PM fluorescence exclusively increased over time to represent 60–70% of the total number of transfected cells (Figure 4B). Under this condition, the number of cells containing intracellular Gag-CFP accumulations always remained low (<12% of the total).

### Endosomal Gag-CFP and Virion Accumulation in HeLa Cells Is Prevented by Inhibiting Endocytosis or Early Endosome Function

**Figure 5:**
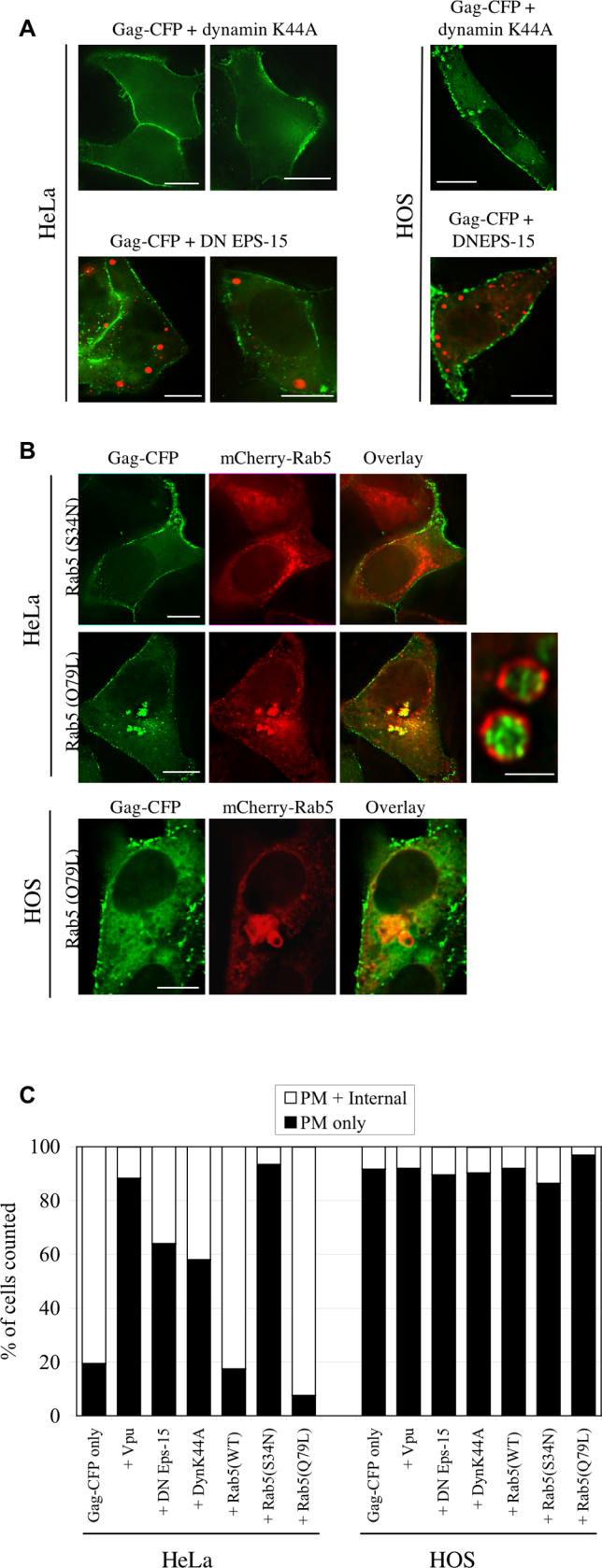
DN Mutants of Cellular Proteins that Mediate PM-to-Early-Endosome Transport Affect Gag-CFP Localization in HeLa, but Not HOS Cells (A) HeLa cells (left panels) or HOS cells (right panels) were transfected with plasmids expressing HIV-1 Gag-CFP (green) and either dynamin K44A (no color) or dnEPS-15-YFP (red). Cells were fixed at 18 h post-transfection and analyzed using deconvolution microscopy. (B) HeLa cells (upper and center panels) expressing HIV-1 Gag-CFP (green), and either DN (S34N) or constitutively active (Q79L) mutants of CherryFP-Rab5a (red), were fixed and images acquired 18 h post-transfection. The far-right-center panel shows an example of how localization of HIV-1 Gag-CFP in swollen CherryRab5aQ79L early endosomes could be resolved as apparently particulate fluorescence within a vesicle lumen. (B, lower panels) shows a similar analysis HIV-1 Gag-CFP localization (green) in the presence of CherryFP-Rab5a(Q79L) in HOS cells. White bars in the micrographs indicate a distance of 10 μm, except in the far-right-center panel of (B) where a distance of 1 μm is indicated. (C) The numbers of HIV-1 Gag-CFP–expressing cells in ten fields exhibiting PM Gag-CFP accumulation only (black bars) or additional intracellular accumulations (white bars) in the presence of the indicated co-expressed cellular proteins was counted, as in Figure 2C, and is plotted as a percentage of the total number of cells with Gag-CFP accumulations.

These results suggested that HIV-1 Gag-CFP accumulates first at the PM of HeLa cells and, in the absence of Vpu, subsequently accumulates in endosomes. Conversely, Vpu expression prevented endosomal localization and preserved the early, primarily PM, distribution of HIV-1 Gag-CFP. This result indicated that Vpu is not required for PM targeting of Gag and suggested the possibility that intracellular accumulations of Gag-CFP might arise via endocytosis of Gag molecules or nascent virion particles that are retained on the PM of HeLa cells in the absence of Vpu. To test this idea, HeLa cells were co-transfected with plasmids expressing HIV-1 Gag-CFP in the presence of dominant inhibitors of endocytosis. Thereafter, cells were examined at 18 h after transfection, at which time intracellular Gag-CFP accumulations would predominate (Figures 2–4). Remarkably, manipulation of PM to early endosome transport pathways dramatically affected HIV-1 Gag-CFP localization in HeLa cells, but not in HOS cells (Figure 5A–5C). Specifically, expression of dominant negative (DN) forms of dynamin (K44A) and EPS-15 (YFP-DN EPS-15), both of which are known to inhibit clathrin-mediated endocytosis [46], significantly reduced the accumulation of Gag-CFP at intracellular sites in HeLa cells (Figure 5A and 5C). Furthermore, expression of CherryFP-Rab5a(S34N), a mutant of Rab5a that fails to bind GTP, blocks endocytic vesicle fusion and inhibits the uptake of both fluid-phase and receptor-mediated endocytic cargo [47–49], and almost completely abolished the appearance of intracellular HIV-1 Gag-CFP in HeLa cells (Figure 5B and 5C). This effect was not observed upon co-expression of Gag-CFP with the wild-type Rab5a protein (Figures 3B and 5C).

Interestingly, expression of a “constitutively active” form of Rab5a(Q79L), which binds GTP but cannot hydrolyze it [47], and which leads to the generation of a swollen early endosomal compartment, also affected Gag-CFP localization (Figure 5B). However, in this case, while the proportion of HeLa cells that exhibited intracellular HIV-1 Gag-CFP accumulation remained similar (Figure 5C), the vast majority of intracellular HIV-1 Gag-CFP co-localized with CherryFP-Rab5a(Q79L) (Figure 5B). This result indicates that Rab5a(Q79L) can trap HIV-1 Gag-CFP in an aberrant early endosome compartment, and presumably prevent further transit to CD63+ late endosomes (Figure 3B). In some cases, these swollen endosomes could be resolved as Rab5a-bound structures containing distinct Gag-CFP accumulations (Figure 5B, far-right panel). Importantly, the wild-type form of Rab5a had no effect on the localization of Gag-CFP in HeLa cells (Figure 3), and neither wild-type nor mutant Rab5a proteins had any effect on Gag-CFP localization in HOS cells. Indeed, Gag-CFP accumulations in HOS cells remained robustly at the PM despite any of the aforementioned manipulations of endocytosis (Figure 5A–5C). Thus, the effect of Vpu expression on HIV-1 Gag localization in HeLa cells can be recapitulated by factors that inhibit endocytosis, and this suggests that Vpu blocks a cellular activity that ultimately results in the endocytosis of Gag molecules or virions.

**Figure 6:**
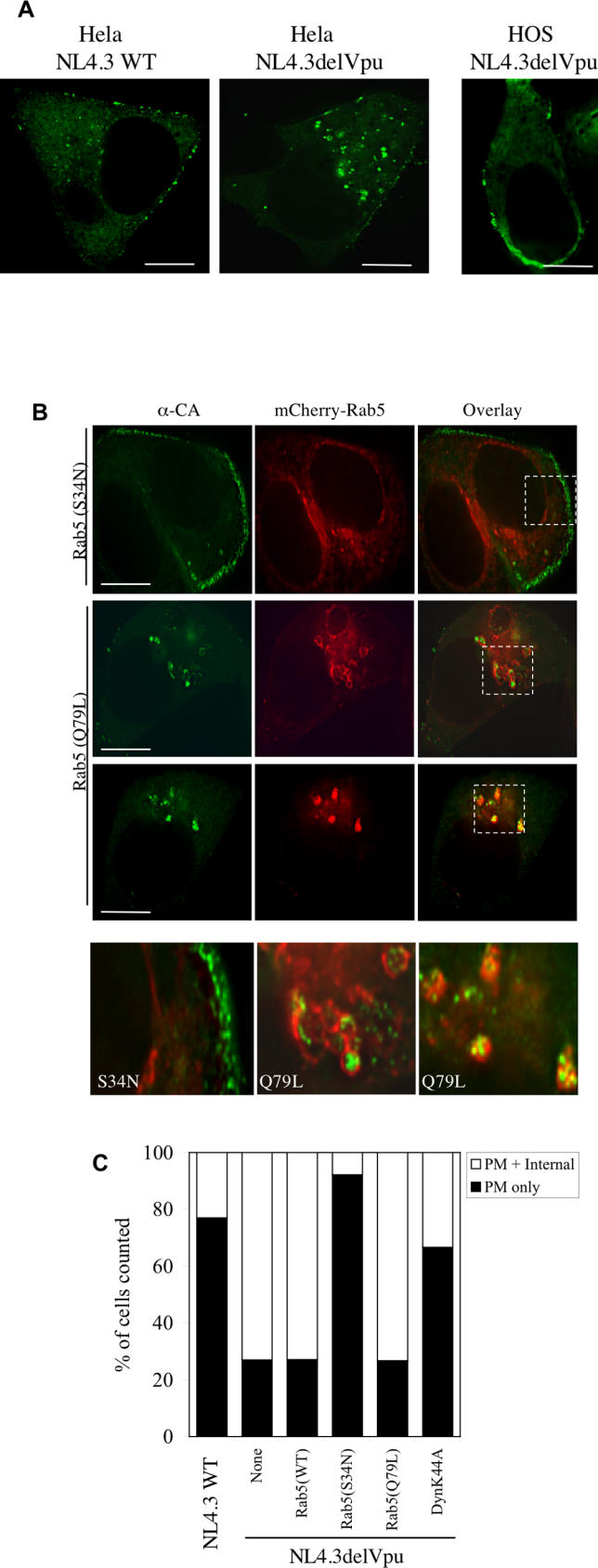
Immunofluorescent Localization of HIV-1 Gag Expressed from a Proviral Construct and Effects of Vpu and Endocytosis Inhibitors (A) HeLa cells (left and center panels) or HOS cells (right panel) were transfected with NL4.3 or NL4.3delVpu proviral plasmids, as indicated. Gag localization was determined by immunofluorescence using an α-CA antibody as described in Materials and Methods. (B) HeLa cells expressing Gag from the NL4.3delVpu proviral plasmid, and either DN (S34N, upper panels) or constitutively active (Q79L center and lower panels) mutants of CherryFP-Rab5a (red), were fixed and subjected to immunofluorescence to detect HIV-1 Gag (green) at 20 h post-transfection. White bars in the micrographs indicate a distance of 10 μm. The lower three images in (B) show expanded views of a portion of the overlay panels (indicated by the dashed squares) to exemplify how HIV-1 Gag and the CherryRab5a proteins are juxtaposed, Note that Gag sometimes appears to be within the lumen of the CherryFP-Rab5a(Q79L)–marked endosomes. (C) The numbers of HIV-1 Gag–expressing cells in ten fields exhibiting PM Gag immunofluorescence only (black bars) or additional intracellular accumulations (white bars) after transfection with NL4.3 or NL4.3delVpu proviral plasmids in the presence of the indicated co-expressed cellular proteins was counted, as in Figure 2C. Data are plotted as a percentage of the total number of cells with visible Gag accumulations.

To verify that the aforementioned effects were not an artifact of the use of Gag-GFP fusion proteins, we repeated several experiments using full-length or Vpu-defective HIV-1 proviral plasmids, coupled with Gag detection by immunofluorescence. As can be seen in Figure 6A, immunofluorescent detection of Gag revealed that it accumulated primarily at the PM of HeLa cells when expressed in the context of an intact proviral plasmid, but was also observed in intracellular compartments when a Vpu-defective proviral plasmid was used. Conversely, Gag accumulations were exclusively at the PM of HOS cells, even when expressed in the absence of Vpu (Figure 6A). Furthermore, Rab5a(S34N) prevented detectable intracellular accumulation of intracellular Gag in HeLa cells, while Rab5a(Q79L) showed significant co-localization with Gag (Figure 6B). Indeed, authentic HIV-1 Gag often appeared to be contained within Rab5a(Q79L)–bound aberrant endosomes (Figure 6B, lower panels). Enumeration of cells exhibiting PM only, or of cells exhibiting additional intracellular accumulations, revealed that authentic Gag molecules behaved in essentially the same way as Gag-GFP in terms of the effects of Vpu and inhibitors of endocytosis.

**Figure 7:**
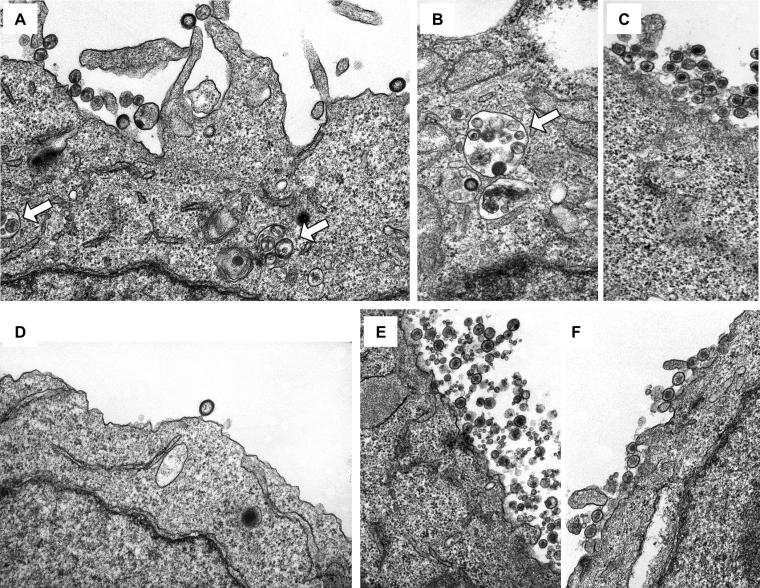
Electron Microscopy Examination of HIV-1 Assembly and Effects of Vpu and Endocytosis Inhibition Hela cells expressing Gag-Pol in the presence or absence of Vpu or Rab5a(S34N) were examined. (A–C) Examples of cells expressing Gag-Pol only, revealing mature particles at both the PM (A and C) and within internal, apparently membrane-bound compartments (arrows in A and B). Immature budding structure are also observed at the PM but only rarely within endosomes. (D) Cells co-expressing Gag-Pol and Vpu with reduced numbers of cell-associated particles, and absence of particles from endosomes. (E and F) Cells co-expressing Gag-Pol and Rab5a(S34N), with exclusively surface-accumulated virions.

In addition, electron microscopy examination of HeLa cells expressing HIV-1 Gag-Pol revealed accumulations of virus particles at both the PM and in intracellular compartments that were apparently membrane-bound (Figure 7A–7C). The majority of these particles had condensed cores, indicating that they were mature, and this was consistent with previous reports that characterized Vpu-defective viruses in T cells [17,18]. Co-expression of Vpu resulted in fewer cell-associated particles (Figure 7D), with no particles detected in intracellular compartments. Similarly, no intracellular particles were observed in cells co-expressing Gag-Pol and Rab5a(S34N), but numerous extracellular particles containing apparently condensed cores were observed. Many of these appeared close to or associated with the extracellular surface of the PM (Figure 7E and 7F).

### Inhibition of Endocytosis Does Not Induce Vpu-Independent HIV-1 Release

**Figure 8:**
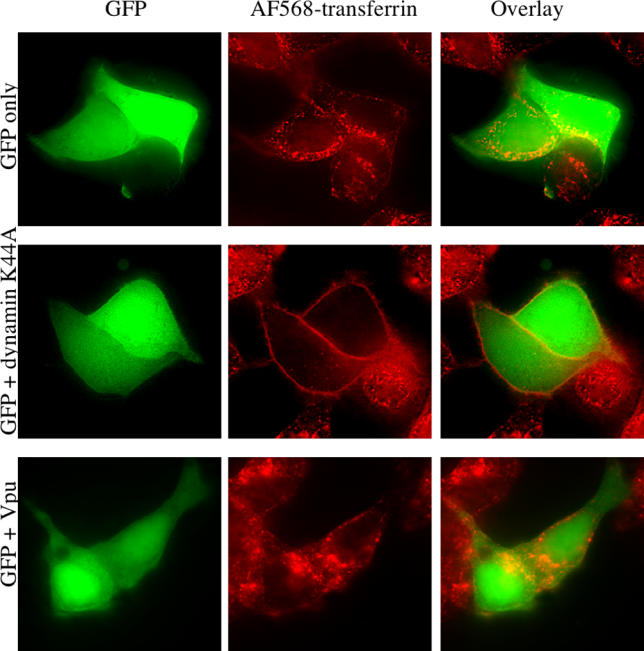
Vpu Expression Has No Effect on Transferrin Endocytosis HeLa cells were transfected with a control plasmid, or with plasmids expressing dynamin K44A, or Vpu together with a GFP-expression plasmid to mark transfected cells. Five hours after transfection, cells were transferred to serum-free medium. After overnight incubation, cells were then washed and incubated in fresh medium containing 5 μg/ml of transferrin conjugated to Alexa-Fluor-568. After 15 min of incubation, cells were fixed and images acquired.

Because inhibition of endocytosis recapitulated the effect of Vpu on Gag localization (Figures 5 and 6), we next asked whether Vpu facilitated virion release from HeLa cells simply by inducing an arrest of endocytosis. Fluorescently labeled Alexa-Fluor-568 transferrin (red) was used as a traceable endocytic cargo and, as can be seen in Figure 7, HeLa cells that were marked by transfection with a GFP-expression plasmid efficiently internalized transferrin (Figure 8). Conversely, co-expression of dynamin K44A (as a control) with GFP abolished detectable transferrin uptake by HeLa cells or by HOS cells, and labeled transferrin remained at the PM of GFP-positive cells (Figure 8; unpublished data). Notably, GFP and Vpu co-expression did not affect transferrin uptake in either HeLa (Figure 8) or HOS cells (unpublished data), indicating that Vpu does not induce a general block in clathrin-dependent endocytosis.

**Figure 9:**
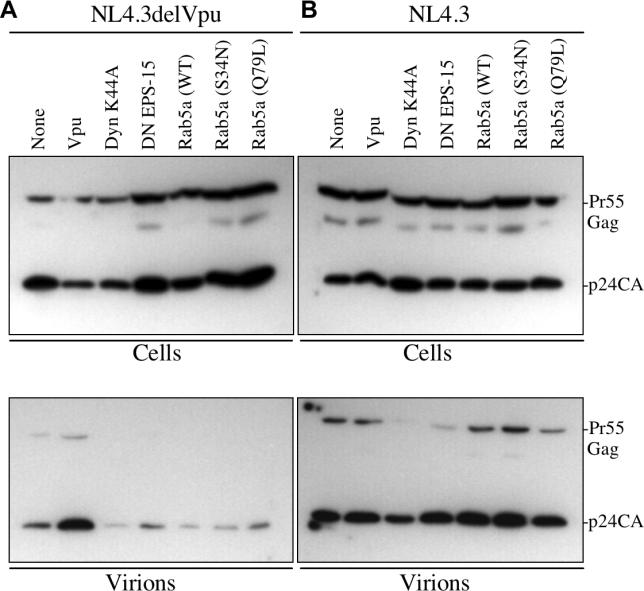
Inhibition of Endocytosis or Early Endosome Function Does Not Restore Vpu-Deficient HIV-1 Release HeLa cells were transfected with NL4.3 (A) or NL4.3delVpu (B) proviral plasmids along with plasmids expressing GFP or Vpu (as controls), dynamin K44A, DN EPS-15, or wild-type and mutant Rab5a proteins. Cell and virus lysates were harvested 48 h post-transfection, and analyzed by Western blotting with an HIV-1 CA-specific monoclonal antibody

Next, we transfected HeLa and HOS cells with NL4.3 or NL4.3delVpu proviral plasmids along with plasmids expressing either Vpu, dynamin K44A, DN EPS-15, or Rab5a mutants. As can be seen in Figure 9, expression of Vpu in *trans* specifically enhanced the release of NL4.3delVpu (Figure 9A and 9B), but none of the aforementioned dominant inhibitors of endocytosis, which prevented intracellular accumulation of Gag and/or particles (Figures 4–6), could recapitulate this activity. Together these results demonstrate that generalized inhibition of endocytosis per se does not explain how Vpu stimulates retrovirus particle release. Moreover, inhibition of endocytosis did not appear to affect Vpu activity since NL4.3 was released more efficiently than NL4.3delVpu, even in the presence of each of the endocytosis inhibitors.

### Endosomal Accumulation of Retroviral Gag Proteins in HeLa Cells Requires a Functional L-Domain

**Figure 10:**
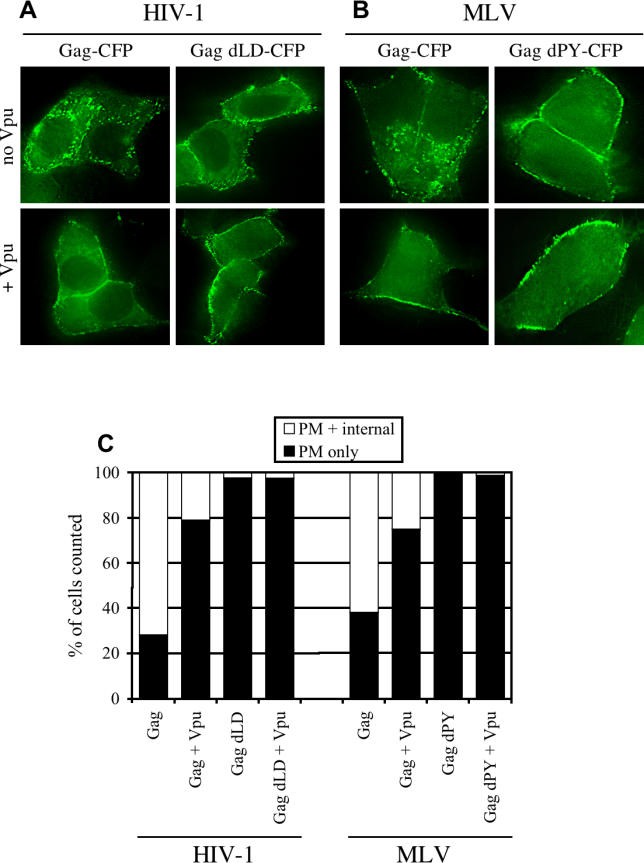
Endosomal Accumulation of HIV-1 and MLV Gag-CFP Requires a Functional L-Domain HeLa cells were transfected with plasmids expressing HIV-1 (A) or MLV Gag-CFP (B), or mutant variants thereof lacking functional L-domains (HIV-1 Gag dLD-CFP and MLV Gag dPY-CFP, respectively) in the presence or absence of co-expressed Vpu, as indicated. The cells were fixed at 18 h post-transfection and images acquired. Representative examples are shown in panels (A) and (B) and a quantitative assessment of Gag-CFP localization, enumerated as in Figure 1, is shown (C). The numbers of HIV-1 Gag-CFP–expressing cells in ten fields exhibiting Gag-CFP accumulation only at the PM (black bars) or at intracellular sites (white bars) were counted, as in Figure 2C, and are plotted as a percentage of the total number of cells with Gag-CFP accumulations.

Retroviral Gag proteins encode small peptide motifs (L-domains) that recruit components of the class E VPS machinery that are required for nascent virion generation via the fission of virion and cellular membranes (reviewed in [43,44]). The block imposed on virion release by L-domain mutation results in the accumulation of nearly complete, but immature, membrane-tethered virions, as opposed to the apparently mature virions that accumulate in the absence of Vpu (Figure 7). Thus, given that intracellular, Vpu-defective virions appear to arise via endocytosis, the block to virion release imposed by the Vpu defect seems to occur subsequently to that imposed by an L-domain defect. Therefore, we next asked whether a functional L-domain and, by inference, either recruitment of class E VPS factors or separation of virion and cell membranes, was required for endocytosis of Gag or nascent virions by HeLa cells. Interestingly, while wild-type HIV-1 Gag-CFP localized to internal structures in HeLa cells, as before, a mutant containing inactivating mutations in both the TSG101-binding PTAP motif [50–52] and the AIP1/ALIX–binding LrsLF motif [53] accumulated exclusively at the PM, even in the absence of Vpu (Figure 10). This observation is consistent with a previous study which showed that the HIV-1 PTAP motif is required for detection of HIV-1 Gag in a biochemically enriched fraction containing endosomes [39].

Interestingly, we also found that a mutant MLV Gag-CFP protein (MLV GagdPY-CFP) that lacks the PPPY motif required for the binding of HECT ubiquitin ligases [54] failed to accumulate at intracellular locations (Figure 10B and 10C), unlike the wild-type MLV Gag-CFP protein. Conversely, a mutant MLV Gag-CFP protein, in which the PPPY L-domain was replaced by the PTAP motif from HIV-1, behaved like wild-type MLV Gag-CFP and localized to internal structures in the absence of Vpu, but was exclusively PM-associated when Vpu was co-expressed (unpublished data). Thus, for both MLV and HIV-1, internalization requires that Gag contains functional L-domain sequences, and either PTAP or PPPY motifs can mediate this effect. This finding suggests that the endocytosis of nascent virions that can occur in the absence of Vpu requires that virion and cell membranes should be discontinuous, or that L-domains recruit factors required for Gag or virion endocytosis.

### Mature, Vpu-Defective Virion Particles Are Tethered to the Surface of HeLa Cells by Protease-Sensitive Factor(s)

**Figure 11:**
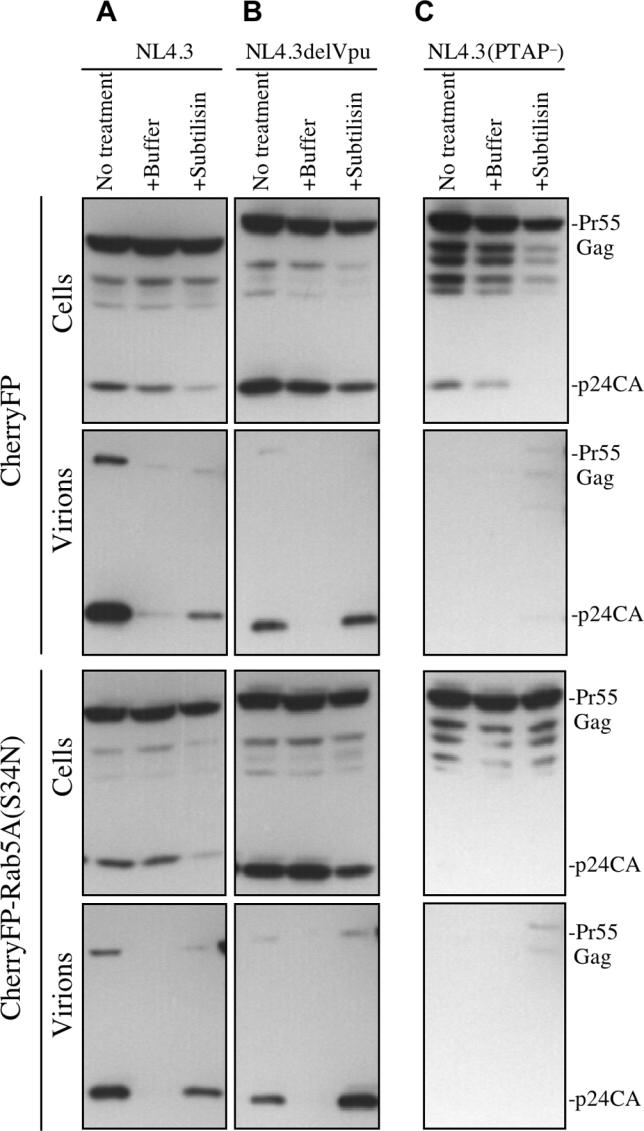
Absence of Vpu Results in Cell-Surface Entrapment of Mature Virions Subtilisin-induced release of mature, Vpu-defective HIV-1 particles from HeLa cells. Hela cells were transfected with NL4.3 (A), NL4.3delVpu (B), or NL4.3 (PTAP^−^) (C) proviral plasmids as indicated, in the presence of CherryFP-Rab5a(S34N) or CherryFP-expression vectors. Cells and supernatants were harvested 48 h post-transfection and analyzed directly (no treatment), while cells in replicate wells were either treated with 1 mg/ml subtilisin (+ subtilisin) or a buffer control (+ buffer) for 15 min at 37 °C. Virions released by buffer or subtilisin and corresponding cell lysates were harvested and analyzed by Western blotting with an anti p24CA antibody as before.

Since the aforementioned experiments (Figures 7 and 10) suggested that the Vpu defect imposes a block to virion release that is subsequent to the action of L-domains, we reasoned that particle endocytosis was likely a downstream consequence of the failed release of fully formed virions from the PM. Implicit in this scenario is some form of post-budding adhesion of nascent virions to the surface of the HeLa cells from which they are derived. To test this notion, HeLa cells were transfected with NL4.3 or NL4.3delVpu proviral plasmids, and the level of particles that were either constitutively released, or were released only following treatment with a protease (subtilisin) was determined (Figure 11). As expected, NL4.3 virions were constitutively released more efficiently than NL4.3delVpu virions. Indeed, far fewer NL4.3 virions remained associated with HeLa cells (in a form that could be released by subtilisin treatment), than the number that were constitutively released (Figure 11A).

In contrast, a significant proportion (at least half) of the virions generated by NL4.3delVpu-transfected HeLa cells were not constitutively released, but could be released following subtilisin treatment (Figure 11B). Thus, the absence of Vpu resulted in a loss of extracellular virion production in favor of an increase in a mature virion population, containing fully processed CA, which could be released from cells by subtilisin. The number of Vpu-defective virions that were released from HeLa cells by susbtilisin treatment was increased when cells co-expressed Rab5a(S34N) to inhibit endocytosis (Figure 11B). Indeed, in this case, the quantity of virions that were released by subtilisin significantly exceeded the quantity that were constitutively released, and actually approached the levels that were constitutively released by the Vpu-competent provirus-transfected cells (Figure 11A and 11B). Notably, subtilisin induced the release of particles that predominantly contained fully processed p24CA and very little p55Gag precursor, suggesting that the particles that were released by protease treatment were fully mature virions.

As a control, the effect of subtilisin on the release of HIV-1 virions lacking a functional L-domain NL4.3 (PTAP^−^) was determined (Figure 11C). In the case of this L-domain mutant, virions are known to remain immature and accumulate at the PM, tethered by a membrane that is contiguous with that of the host cell. Importantly, subtilisin treatment did not increase the release of NL4.3 (PTAP^−^) virons, irrespective of Rab5(S34N) co-expression. This finding indicates that subtilisin treatment does not compromise the integrity of the PM and that Vpu-defective virions differ from late-budding domain mutant virions in that the former are tethered to the PM by one or more protease-sensitive factor(s), and not by continuity between the virion envelope and the PM.

## Discussion

Although Vpu can strongly induce retrovirus particle release from cells, we, and others, have not observed co-localization, or any other form of interaction, between retroviral Gag proteins and Vpu (unpublished data). Vpu can localize to intracellular membranes of the endoplasmic reticulum, golgi, and endosomal structures, as well as the PM [17,55,56]. While ER localization is required for CD4 down-regulation, and may be required for enhancement of virus release [57], the site at which events relevant to particle release occur is not unambiguously established. A recent study that showed that expression of DN Rab11a or MyosinVb can inhibit HIV-1 release in the presence of Vpu suggests that a functioning recycling endosomal compartment is required [56]. Since Vpu localizes to sites distinct from those at which retroviral particles assemble, and from which they are released, it seems likely that the virus-release function of Vpu results from indirect effects on the host cell, rather than on specific components of the virion. Our findings are consistent with this notion, particularly when viewed alongside a recent study that strongly suggested that Vpu overcomes a host cell–specific restriction to retrovirus particle release in human cells [28].

The effect of Vpu on virus release varied significantly among human cell lines, and HeLa cells proved particularly useful for studying this property, because Vpu stimulated both HIV-1 and MLV release by about 10- to 20-fold from these cells [18]. Conversely, human HOS cells served as a useful control, because efficient HIV-1 and MLV release was completely independent of Vpu, as was previously shown to be the case in AGM cells [28]. By observing the localization of Gag-CFP fusion proteins as well as authentic Gag proteins in HeLa and HOS cells, we found that Vpu prevented the accumulation of HIV-1 and MLV Gag in HeLa cell endosomal compartments, and instead constrained Gag accumulation to the PM. In the absence of Vpu, accumulation of Gag at intracellular sites required a functional L-domain and was blocked by inhibitors of clathrin-dependent endocytosis (DN dynamin and EPS-15) or the function of early endosomes (DN Rab5a). Our observations strongly suggest that accumulations of retroviral Gag proteins and virion particles in endosomes, observed here and previously in the context of defective Vpu [17,18,30], arise from endocytosis of completely assembled virions from the PM. Consistent with this notion, an aberrant early endosomal compartment that is induced and marked by Rab5a(Q79L) appeared to contain the majority of intracellular HIV-1 Gag in the absence of Vpu.

It is important to note that while these experiments document a PM-to-endosome trafficking route for two retroviral Gag proteins, they do not address how Gag first achieves PM localization. Indeed, several groups have suggested that retroviral Gag molecules and/or completely assembled particles traffic to the PM and/or leave the cell via an intermediate step that involves Gag localization and even particle formation in endosomes [33–40]. While the existence of a late endosome-to-PM trafficking route for HIV-1 particles or assembly intermediates has gained wide acceptance [58], the precise method by which Gag moves to the PM in non-macrophage cell types remains somewhat ambiguous. The findings reported herein highlight the need to distinguish between Gag molecules that are directly targeted to endosomal membranes and those that arrive in endosomes as a consequence of virion endocytosis from the PM. Given the dramatic effects of the host-cell type, the presence or absence of Vpu, and the effect of Gag expression level on steady-state Gag distribution [59,60], it is perhaps unsurprising that there is wide discordance among studies that have determined this superficially simple parameter.

**Figure 12:**
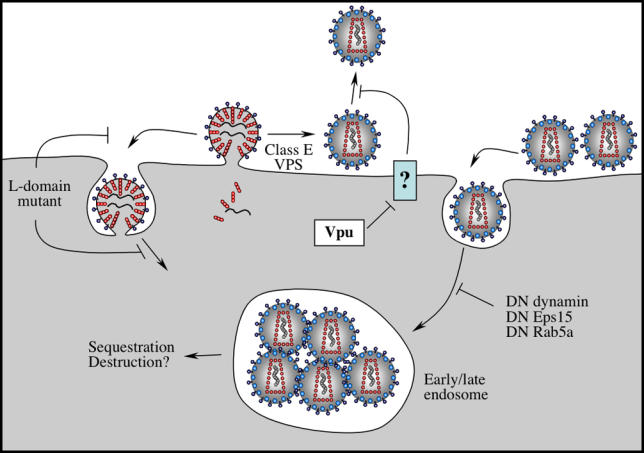
Model for the Role of Vpu in Retroviral Particle Release Retroviral Gag molecules targeted to the PM (by unknown mechanisms) require the recruitment of class E VPS factors to enable budding of mature virions. At this point, nascent viral particles are subject to an unknown, subtilisin-sensitive, host-cell type-specific restriction (depicted as a question mark) that results in their retention at the cell surface. Subsequently, endocytosis by a dynamin-, EPS-15-, and Rab5a-dependent process result in intracellular accumulation in early/late endosomes, where they may be simply sequestered, or perhaps destroyed, in lysosomes. Vpu overcomes this restriction, leading to more efficient release of virions from the cell surface. In contrast, inhibition of endocytosis by DN mutants of dynamin, EPS-15, or Rab5a prevents the accumulation of virions in endosomes, but does not enhance virion release, suggesting Vpu inhibits a specific host-restriction activity, rather than imposing a generalized inhibition on endocytosis.

While Vpu appeared to prevent endocytosis of nascent retrovirus particles, it did not appear to inhibit endocytosis in a general sense, as judged by transferrin-uptake assays. Moreover, generalized inhibition of endocytosis prevented intracellular accumulation of HIV-1 and MLV Gag but could not substitute for Vpu function. Therefore, we propose that endocytosis of nascent virions is a downstream consequence of absent Vpu function, and that Vpu inhibits an activity that prevents the release of completely assembled virions from the PM (Figure 12). While the nature of this putative host-restriction activity is unknown, its existence is the simplest explanation for our findings and is completely consistent with earlier studies on Vpu which showed that Vpu-defective HIV-1 particles accumulate at the cell surface as well as in intracellular vacuoles as mature virions [17,18]. One possibility is that virions remain attached to cells via incorporation of cellular proteins with adhesive properties into the viral membrane. That mature, Vpu-defective, virions can be released from the cell surface by protease treatment, particularly when endocytosis is inhibited, is entirely consistent with this notion. Indeed, a variety of cell-surface proteins are well known to be incorporated into the HIV-1 virion membrane [61]. This scenario has several conceptual attractions. First, an analogous situation exists in other virus families. Many viruses that bind to sialic acid molecules for cell entry require a neuraminidase enzyme to release nascent virions from the cell surface [62]. Second, Vpu is known to target two cell-surface receptors, CD4 [8] and MHC class I [63], for destruction by proteosomes. Thus, Vpu-mediated destruction of other specific cell-surface factors with adhesive properties could conceivably enhance virion release.

Variations on this scenario are also possible. Because endosomal localization of Vpu-defective HIV-1 Gag and MLV exhibits a requirement for a functional L-domain, we currently favor the interpretation that discontinuity of virion and cell membrane is required for particle endocytosis. However, it is also possible that whatever restriction factors impose Vpu dependence and induce Gag endocytosis, they are themselves indirectly recruited by L-domains. Indeed, L-domain motifs are known to recruit ubiquitin ligases [54] and/or class E VPS factors to sites of virus budding [51–53,64]. Since other class E VPS factors (for example Hrs [65] and AIP-1/ALIX [66]) are known to also regulate endocytic traffic, it is entirely plausible that Vpu may prevent the L-domain–dependent recruitment of endocytosis-inducing or virus release–inhibiting factors to sites of virion assembly. In our view, however, this model is less attractive, because it is more difficult (albeit not impossible) to reconcile with the observation that the Vpu defect induces accumulation of fully assembled mature virions at the cell surface [17,18] (Figures 8 and 11) .

In conclusion, the results presented herein strongly suggest that that Vpu prevents entrapment of nascent retroviral particles at the cell surface and, as a consequence, subsequent transport into early and late endosomal compartments. The absence of a such a pathway in HOS cells, and the finding that generalized inhibition of endocytosis “corrects” Gag localization but does not enhance release, strongly suggests that specific host-cell factors that are targeted by Vpu, but otherwise prevent efficient release of widely divergent retroviruses from the PM, exist in some human cell types. Clearly, the identification of Vpu target factors that exhibit such properties will be required to definitively show whether or not such a model is correct.

## Materials and Methods

### Cells and plasmids.

HeLa, HeLa-TZM, HOS, and Vero cells were maintained in Dulbecco's Modified Eagle Medium supplemented with 10% fetal calf serum and gentamycin. The expression vector pcDNA-Vphu, expressing a codon-optimized HIV-1 Vpu protein from the NL4.3 strain, was provided by K. Strebel (National Institutes of Health, Bethesda, Maryland, United States) through the NIH AIDS Research and Reference Reagent Program (Germantown, Maryland, United States). A derivative of this plasmid in which the Vpu TM domain was replaced by that of the human CD8 molecule was constructed using an overlapping PCR-based approach. Similarly, a Vpu-defective NL4.3-based proviral plasmid (NL4.3delVpu) was constructed by introduction of a frame shift and replacement of the initiating codon of Vpu with a *Bam*H1 site using an overlapping PCR-based approach. To express Vpu with C-terminal HA and YFP tags, wild-type HIV-1 NL4.3 Vpu sequences were amplified by PCR and inserted as an EcoR1-Xho1 fragment into the mammalian expression plasmids pCR/HA and pCR/YFP that have previously been described [67]. Plasmids expressing DN dynamin I (K44A) and DN EPS-15 (δUIM) were kindly provided by M. Caron (Duke University, Durham, North Carolina, United States) and S. Polo (FIRC Institute for Molecular Oncology, Milan, Italy), respectively. Human Rab5a was amplified from human placental cDNA and inserted as an EcoR1-Xho1 fragment fused at its 5′ end to a CherryFP cDNA (provided by R. Tsien, University of California San Diego, San Diego, California, United States) in a pCR3.1-based plasmid, to express a CherryFP-Rab5a fusion protein. Mutants (S34N and Q79L) were derived using overlapping PCR-based methods. Plasmids expressing wild-type and mutant Gag-CFP fusion proteins, namely pCR/HIV-1Gag-CFP [60], pCAGGS/MLVGag-CFP, pCAGGS/MLVGag(dPY)-CFP, and pCAGGS/MLVGag(PTAPP)-CFP have been described previously [54,68]. A derivative of pCR/HIV-1Gag-CFP lacking the bipartite L-domain in p6 (PTAP and LRSLF were mutated to LTAL and LRSPS, respectively) was generated using overlapping PCR methods.

### Virus-release assays.

HeLa, HOS, or Vero cells were seeded at 1 × 10^5^ cells/well of a 24-well plate and were transfected with a total of 1 μg of DNA/well consisting of 800 ng of NL4.3, NL4.3delVpu, or MLV Gag-Pol, together with 200 ng of Vpu, dynamin, EPS-15, or Rab5a-expression vectors, using LipoFectamine 2000 (Invitrogen, Carlsbad, California, United States). After 48 h, the cell supernatants were harvested, filtered (0.2 μm), and layered (500 μl) on to 400 μl of 20% sucrose in PBS and centrifuged at 20,000 *g* for 90 min at 4 °C. Pelleted virions were resuspended in SDS-PAGE loading buffer, as were corresponding cell lysates. Virion and cell lysates were separated on 12% SDS-PAGE gels (Bio-Rad, Hercules, California, United States), and proteins were transferred to nitrocellulose membranes which were then probed with HIV-1 or MLV CA-specific monoclonal antibodies (183-H12-5C or D175, respectively) and with the appropriate HRP-conjugated goat secondary antibodies.

In experiments where subtilisin-induced release of virions was attempted, HeLa cells were transfected with NL4.3, NL4.3delVpu, or NL4.3 (PTAP^−^) in the presence or absence of co-expressed Rab5a(S34N). Viral supernatants were harvested as above, and parallel wells were washed once with PBS and then either directly harvested or, alternatively, incubated for 15 min at 37 °C in 100 μl of Tris/HCl (pH 8.0), 150 mM NaCl, 5 mM CaCl_2_ with or without the addition of subtilisin (1 mg/ml). The reaction was then stopped with 0.5 ml DMEM/FCS containing 5 mM PMSF. The supernatants were filtered (0.2 μm) and the virions pelleted through sucrose as described above.

Infectious virus release was determined by inoculating HeLa-TZM cells (CD4 and CCR5+), which contain a *lacZ* reporter gene under the control of an HIV-1 LTR. Sub-confluent monolayers of HeLa-TZM cells were infected for 4 h, washed, and β-galactosidase activity was determined 48 h later using GalactoStar reagent (Tropix, Bedford, Massachusetts, United States), as per the manufacturer's instructions. In experiments where MLV infectious titers were measured, an HIV-1 Tat–expressing, packageable MLV vector (LXSN/Tat) and VSV-G–expression vectors were included in the particle-generating transfection mixture.

### Microscopy.

Cells were seeded on 3.5-cm, glass-bottomed dishes coated with poly-l-lysine (Mattek, http://www.mattek.com). The following day, they were transfected with 1.5 μg of HIV-1 or MLV Gag-CFP–expression vectors and 0.5 μg of empty pCR3.1 or expression vectors encoding Vpu, dynamin K44A, DN EPS-15, or Rab5a, using polyethylenimine. Cells were fixed at 16 to 18 h after transfection, or at the time points indicated in the text, using 4% paraformaldehyde for 10 min. Cells were then observed by deconvolution microscopy using an Olympus IX70-based Deltavision microscopy suite (Tokyo, Japan) and a 60× objective, and analyzed using SoftWorx software (Applied Precision, Issaquah, Washington, United States). CFP, YFP, and CherryFP fused proteins were excited at 436, 500, and 575 nm respectively. Similar approaches were used to determine the localization of Gag proteins expressed by the NL4.3 and NL4.3delVpu proviral plasmids in the presence or absence of co-expressed CherryFP-Rab5a fusion proteins. However, in this case, cells were permeabilized after fixation with 1% TritonX-100 and then stained by sequential incubations with an α-HIV-1 CA monoclonal antibody (183-H12-5C) and an Alexa-Fluor-495–labeled α-murine IgG conjugate. For electron microscopy studies, HeLa cells were transfected with plasmids expressing HIV-1 Gag-Pol (pSYNGP) in the presence or absence of plasmids expressing Vpu or CherryFP-Rab5a(S34N). Cells were processed and examined by electron microscopy as described previously [64,69].

### Transferrin-uptake assays.

HeLa cells were seeded on poly-l-lysine–coated glass-bottomed plates (Mattek) and transfected the following day with a total of 1 μg of 1:1 mixtures of pCR/GFP with either pCR/HA, pcDNA-Vphu, or dynamin K44A-expression vectors for 5 h using polyethylenimine. The cells were then washed and serum-starved overnight. The cells were then incubated for 30 min on ice in serum-free medium containing 5 μg/ml of Alexa-Fluor-568–conjugated transferrin. The medium was replaced and the cells were shifted to 37 °C for 15 min, then washed and fixed in 4% paraformaldehyde and observed by deconvolution microscopy as described above.

## Abbreviations

AGM: African green monkey
CA: capsid
CFP: cyan fluorescent protein
CherryFP: cherry fluorescent protein
DN: dominant negative
GFP: green fluorescent protein
HIV: human immunodeficiency virus
L-domain: late-budding domain
MLV: murine leukemia virus
PM: plasma membrane
TM: transmembrane
VPS: vacuolar protein-sorting
Vpu: human immunodeficiency virus type-1 viral protein U
